# Kimura disease forming a human polyomavirus 6–negative parotid gland nodule with prominent squamous metaplasia in a young female: A case report

**DOI:** 10.1016/j.radcr.2023.02.027

**Published:** 2023-03-16

**Authors:** Kenji Yorita, Tatsuya Fujii, Toshitaka Nagao, Ichiro Murakami, Yumiko Hashida, Tomonori Higuchi, Masanori Daibata, Makoto Toi, Yoshiyuki Ayada, Takuro Igawa

**Affiliations:** a Department of Diagnostic Pathology, Japanese Red Cross Kochi Hospital, 1-4-63-11, Hadaminami-machi, Kochi-shi, Kochi, 780-8562, Japan; b Department of Diagnostic Pathology, Kochi University Hospital, Kohasu, Oko-cho, Nankoku-shi, Kochi, 783-8505, Japan; c Department of Otorhinolaryngology, JA Kochi Hospital, 526-1, Nakano, Aza, Myouken, Nankoku-shi, Kochi, 783-8509, Japan; d Department of Anatomic Pathology, Tokyo Medical University, 6-1-1 Shinjuku, Shinjuku-ku, Tokyo, 160-8402, Japan; e Department of Pathology, Kochi Medical School, Kochi University, Kohasu, Oko-cho, Nankoku-shi, Kochi, 783-8505, Japan; f Department of Microbiology and Infection, Kochi Medical School, Kochi University, Kohasu, Oko-cho, Nankoku-shi, Kochi, 783-8505, Japan; g Department of Pathology, Dentistry and Pharmaceutical Sciences, Okayama University Graduate School of Medicine, 2-5-1, Shikata-cho, Kita-ku, Okayama, 700-8558, Japan

**Keywords:** Kimura disease, Parotid gland, Pathology, Differential diagnosis, Human polyomavirus 6

## Abstract

A case of an asymptomatic 19-year-old woman with Kimura disease presenting with a nodule in the right parotid gland is presented. She had a medical history of atopic dermatitis and noticed a mass on her right-side neck. Cervical lymphadenopathy was clinically diagnosed. The initial management plan was to observe the lesion, which had enlarged from 1 cm to 2 cm in diameter 6 months later. An excisional biopsy was performed, and the pathology confirmed an eosinophil-containing inflammatory parotid gland lesion with many squamous nests and cysts, mimicking a parotid gland tumor. High serum immunoglobulin E levels, peripheral blood eosinophilia, and pathological and genetic diagnoses confirmed Kimura disease. The lesion tested negative for human polyomavirus 6. No recurrence was observed 15 months after the biopsy. The prognosis of Kimura disease without human polyomavirus 6 infection may be favorable; however, further validation of this hypothesis is required as only 5 or 6 cases of Kimura disease have been evaluated for this viral infection. Proliferative squamous metaplasia occurring in parotid gland lesions of Kimura disease is rare and may complicate the diagnostic imaging and pathological diagnosis.

## Introduction

Kimura disease (KD) is a chronic inflammatory disease that commonly affects young Asian males. The clinical features of KD are characterized by blood eosinophilia, increased serum immunoglobulin E (IgE) levels, and head and neck lesions involving subcutaneous tissue, salivary glands, and/or single or multiple regional lymph nodes [1]. In salivary glands, the parotid glands are the main sites of KD. Proliferative squamous metaplasia in parotid gland lesions of KD is rare; only one case has been reported [2]. Recently, human polyomavirus 6 (HPyV6) was identified as a possible causative agent of KD; however, only 5 or 6 cases of KD have been evaluated for this viral infection [3,4]. We clinically, histopathologically, and genetically present the case of a young woman with KD showing a nodule in the right parotid gland. This patient's case was interesting because the KD lesion clinically mimicked cervical lymphadenopathy and histologically mimicked a salivary gland tumor with prominent squamous metaplasia. In addition, we evaluated whether HPyV6 DNA was present in the KD lesion.

## Case report

### Clinical findings

**Fig. 1:**
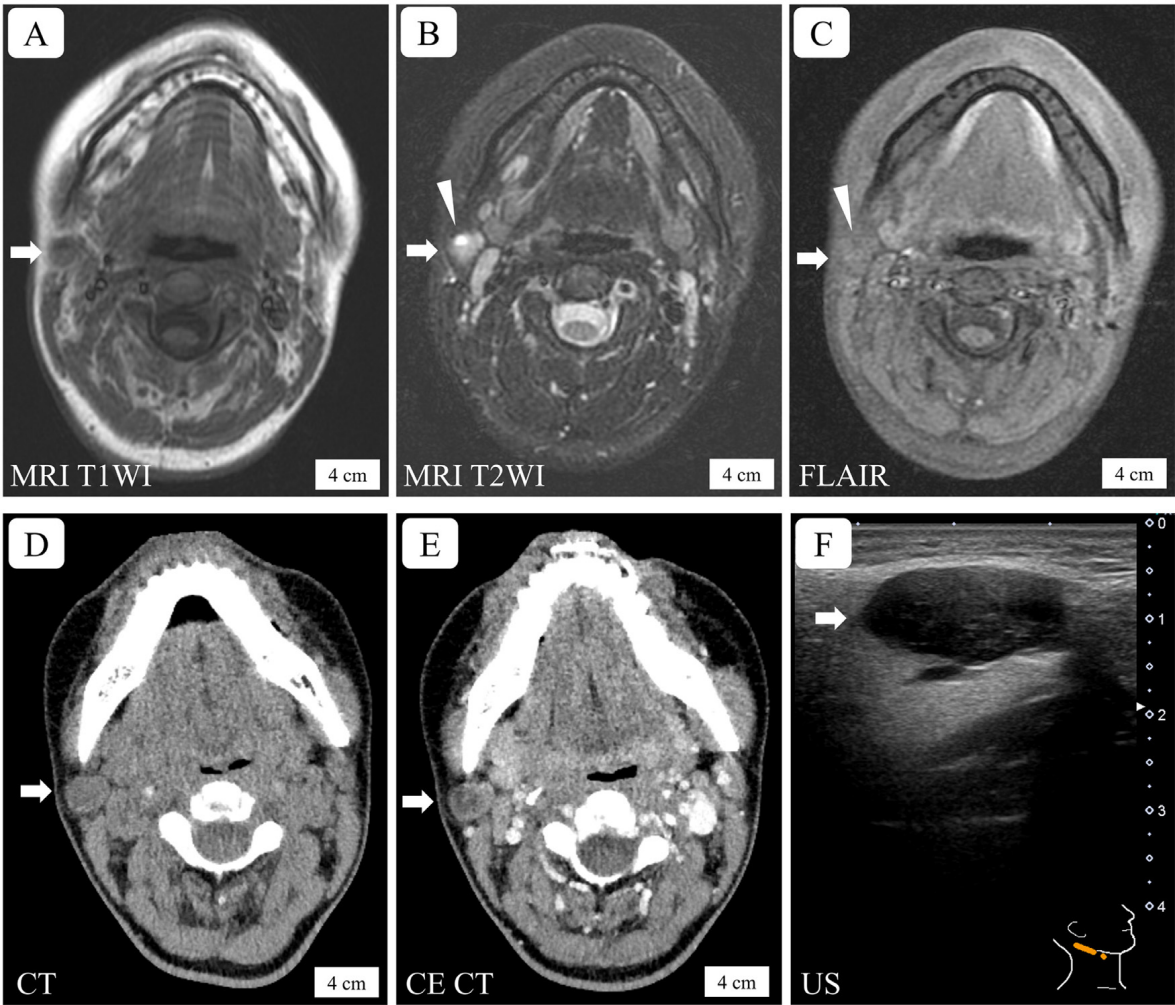
Radiological images of a parotid gland nodule in a young woman with Kimura disease. (A–B) Magnetic resonance images show that the mass has isointensity on a T1-weighted image (A, arrow) and hyperintensity on a T2-weighted image (B, arrow). (C) A fluid-attenuated inversion-recovery image shows an isointense nodule (arrow) with a small low-intensity lesion (arrowhead), which corresponds to a round hyperintense lesion (B, arrowhead) in the nodule on the T2-weighted image. (D–E) Computed tomography images show the nodule is isodense on precontrast imaging (D, arrow) and demonstrates mild heterogeneous enhancement on post-contrast imaging (E, arrow). (F) Ultrasonography performed just before the excisional biopsy demonstrates that the nodule (arrow) is oval and homogeneously hypoechoic. The lengths of the scale bars are shown in the photos. MRI T1 (T2) WI: magnetic resonance imaging T1 (T2) weighted image, FLAIR: fluid-attenuated inversion-recovery, CT: computed tomography, CE CT: contrast-enhanced CT, US: ultrasonography.

A 19-year-old nulliparous woman was referred to our hospital because of a swollen, slightly painful mass in the submandibular region of the right-side neck. She had a history of atopic dermatitis but no history of surgery or treatment for any other disease. She was not taking any medication. The patient's family history and vital signs were unremarkable. The right-side neck lesion was smooth, well-demarcated, firm, and nontender. Serological and urinary test results were unremarkable; T-SPOT, an assay for tuberculosis, and tests for squamous cell carcinoma antigen and soluble interleukin 2 receptor were within normal limits. Magnetic resonance imaging (MRI) showed a nodular lesion measuring 1 cm in diameter near the lower pole of the right parotid gland (Fig. 1A–D). The nodule showed isointensity on T1-weighted images (T1WI) and hyperintensity on T2WI (Fig. 1A–C). The nodule appeared to have a pooling of fluid because T2WIs (Fig. 1B) showed that the mass had a round higher-intensity lesion that corresponded to the hypointensity site on the fluid-attenuated inversion-recovery image (Fig. 1C). Computed tomography (CT) images showed that the lesion was isodense on precontrast CT images (Fig. 1D) and had heterogeneous enhancement on postcontrast CT images (Fig. 1E). Fine-needle aspiration cytology (FNAC) of the neck lesion showed the presence of lymphocytes and tangible-body macrophages; however, no abnormal cells suggestive of neoplasia were found. Therefore, a swollen lymph node in the neck was considered. The initial management strategy involved observation of the lesion. The lesion was enlarged to 1.7 cm diameter at the 3-month follow-up. A second FNAC procedure was performed; however, cytological evaluation was not possible because few cells were observed. After six months of follow-up, ultrasonography confirmed the oval hypoechoic mass had increased to 2 cm in diameter (Fig. 1F). An excisional biopsy was performed to evaluate the neck lesion. During the surgery, the lesion was found to be slightly connected to the right parotid gland.

Following the pathological diagnosis described below, Sjögren syndrome, immunoglobulin G4-related disease (IgG4-RD), human immunodeficiency virus (HIV) infection, and KD were considered as differential pathological diagnoses. Serum levels of Sjögren syndrome anti-SSA, anti-SSB, and anti-HIV 1/2 antibodies were within normal limits or negative. The serum IgG4 level was mildly elevated (144 mg/dL, normal range: 11–121 mg/dL); however, no swollen masses or organs were observed in the lacrimal and salivary glands, except for the right-side neck mass. In addition, peripheral blood eosinophilia (888/μL, 10.5% of total white blood cells, normal range: 70–440/μL) and high serum IgE (424.5 IU/mL, normal range: ≤ 170 IU/mL) were found. With follow-up alone, she was disease-free 15 months after the biopsy; the patient is currently on follow-up. The patient provided written informed consent for the publication of this case report.

### Pathological and genetic findings

**Fig. 2:**
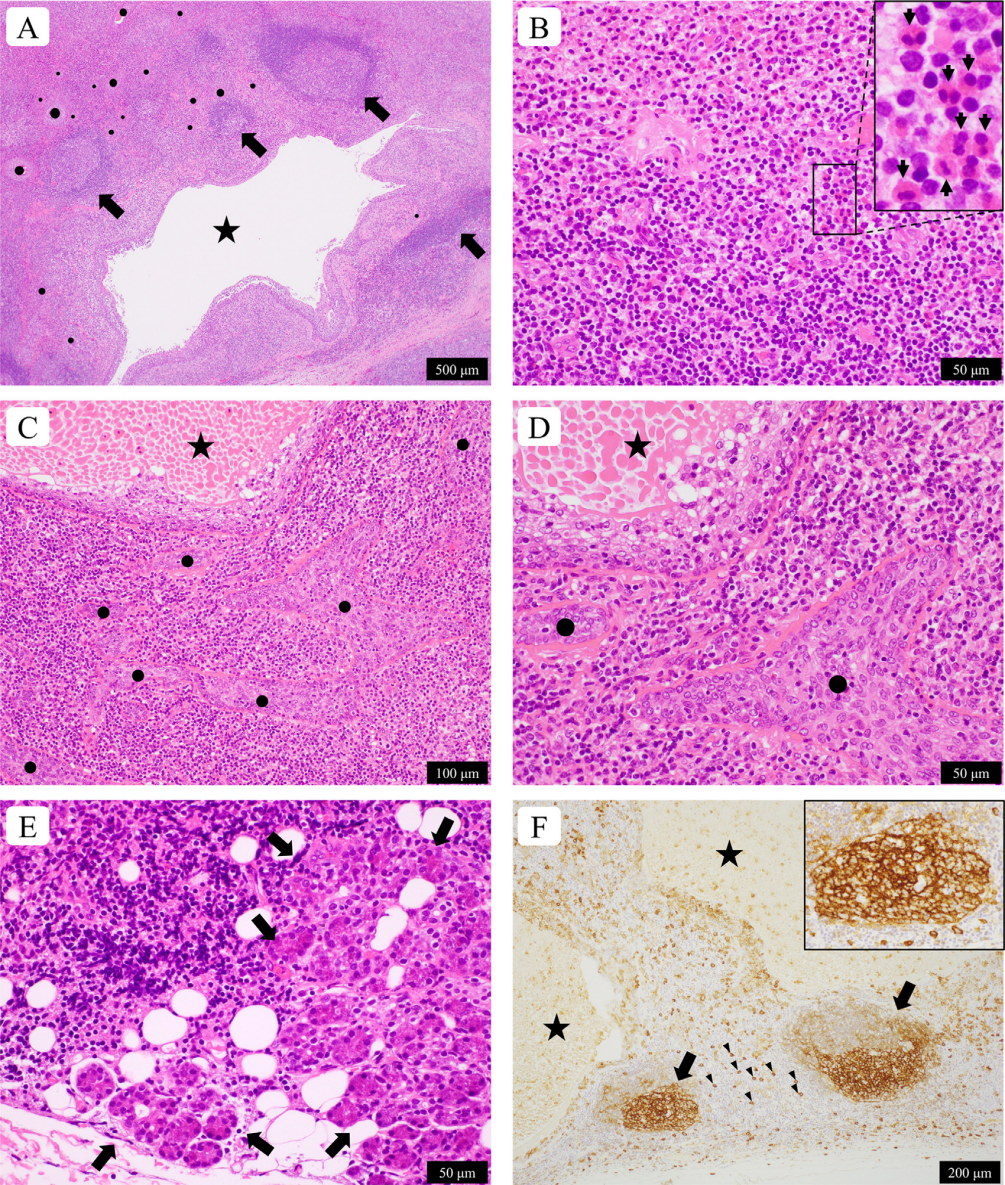
Histological and immunohistochemical images of a parotid gland mass in a young woman with Kimura disease. (A) An inflamed tissue with lymphoid follicles (arrows), squamous nests (dots), and a cystic lesion (star) is seen. (B) A high-magnification photo shows that the main inflammatory cells are lymphocytes and eosinophils (arrows in the inset image). (C–D) Squamous nests (dots) and a squamous cyst (star) are embedded in the inflamed stroma. No atypia is seen in the epithelium (D). (E) Serous glands (arrows) suggestive of parotid gland tissue are present at the periphery of the lesion. (F) Immunohistochemistry using anti-immunoglobulin E antibodies shows positive germinal centers (arrows) and many scattered positive cells (some are indicated by arrowheads) in the inflamed lesion. A reticular pattern of immunoglobulin E staining in germinal centers is seen in the magnified photo (inset). Stars indicate squamous cysts. A–E: hematoxylin and eosin staining. The lengths of the scale bars are shown in the photos.

Macroscopically, the lesion was 2.1 × 1.5 × 1.2 cm. Microscopically, the demarcated nodule had no circumscribed fibrous border and consisted of epithelial tissue and various inflammatory cells, including lymphocytes, eosinophils, plasma cells, and macrophages (Fig. 2A and B). Reactive lymphoid follicles were observed (Fig. 2A), and eosinophilic infiltrates were focally observed (Fig. 2B). Plasma cells were scattered. Many squamous nests and cysts without hyperkeratosis and granular layers were embedded in the inflammatory lesion (Fig. 2A, C, and D), and ductular structures were found on the periphery of the lesion. The epithelial cells had swollen nuclei; however, pleomorphism was absent, and mitosis was rarely observed. Serous glands suggestive of normal parotid glands were observed in the peripheral area of the resected mass (Fig. 2E). Thus, the lesion appeared to have occurred in the right parotid gland.

The initial histological diagnoses included chronic sialadenitis with reactive epithelial proliferation, salivary gland tumor (lymphadenoma, mucoepidermoid carcinoma), and extranodal marginal zone lymphoma of mucosa-associated lymphoid tissue (MALT lymphoma). Chronic sialadenitis with cystic lesions suggested some differential diagnoses, including Sjögren syndrome, HIV infection, and Epstein–Barr virus-associated lymphoepithelial cysts. IgG4-RD was listed as a differential diagnosis should it be necessary.

Mucinous epithelial cells were absent in sections stained using Alcian blue and periodic acid–Schiff with diastase treatment. Immunohistochemically, the squamous nests or cysts were positive for p63 and cytokeratin 5 (CK5) and negative for CK7. The ductal epithelium was confirmed by the presence of a 2-layered structure of CK7^+^/p63^−^ luminal cells and p63^+^/CK7^−^ basal cells. A reverse transcription polymerase chain reaction test did not detect a CRTC1/3–MAML2 fusion gene. The infiltrating cells were mainly CD3-positive lymphocytes with associated CD20-positive lymphoid follicles. No marginal-zone proliferation of atypical B lymphocytes was observed, and no light-chain restriction was detected by immunohistochemistry or in situ hybridization (ISH). Epstein–Barr virus-encoded small RNA1 (EBER1) tested negative by ISH. IgG4-positive plasma cells were observed at 25/high power field and the IgG4/IgG ratio was approximately 41%. However, a dense lymphoplasmacytic infiltrate, storiform fibrosis, and obliterative phlebitis were absent.

Based on the clinicopathological and genetic findings, the differential diagnoses mentioned above were unlikely. Considering that eosinophilic infiltrates were found in the parotid gland lesion, KD could be a histological diagnosis. A histological review of the specimen confirmed proteinaceous materials in the germinal centers and a few multinucleated giant cells, suggesting Warthin–Finkeldey giant cells; however, eosinophilic abscesses and follicles involving eosinophils were not observed. Immunohistochemically, the germinal centers tested positive for IgE (Fig. 2F) and negative for IgG, IgM, and IgA. In addition, many IgE-positive cells were observed in the interfollicular areas (Fig. 2F). Histiocytic or epithelioid endothelium was not observed in the capillaries of the lesion. Thus, based on clinical information (high serum immunoglobulin E levels and peripheral blood eosinophilia), KD was considered.

A possible causative agent, HPyV6 [3,4], was evaluated. A quantitative polymerase chain reaction did not detect HPyV6 DNA among the DNA extracted from the formalin-fixed paraffin-embedded KD lesion. The method of the detection of HPyV6 DNA was followed as described previously [3].

## Discussion

This case report presents a young female with KD showing unilateral parotid gland involvement. The lesion in this case was clinically identified as cervical lymphadenopathy. KD usually affects the subcutaneous tissue and regional lymph nodes, predominantly in the head and neck region [1]. Interestingly, our case showed that only the right parotid gland appeared to be a site of KD. Salivary gland involvement in KD is rare in non-Asian patients but is seen occasionally in Asian patients [1].

In our case, a preoperative diagnosis of KD was difficult because the lesion clinically showed a single and painful swollen lymph node of the neck, suggesting infectious lymphadenitis [5]. In this case, KD might have been ruled out by sex; 86% of patients with KD are male [1]. Moreover, the lesion in this case was unusual for KD-related lymphadenopathy; 89% of KD-related cervical lymphadenopathy occurs only in the posterior cervix [6].

Except for cystic changes, the CT and MRI findings in our case were typical for non-nodal KD lesions of the head and neck [7], although radiological findings specific to KD have not been reported. In our case, the parotid gland lesion involved in KD was pathologically unique because of the presence of various-sized squamous cysts. One of these cysts was recognized radiologically as a pooling of fluid on MRI images. Cystic changes in KD parotid gland lesions have not been reported and may make the diagnostic imaging of KD in the parotid glands more difficult. Therefore, a preoperative diagnosis of KD appeared to be impossible in our case.

Key histological and immunohistochemical features of KD include reactive follicular hyperplasia, eosinophilic infiltrates (eosinophilic microabscess and eosinophils within the germinal centers), eosinophilic proteinaceous deposits within germinal centers, Warthin–Finkeldey giant cells, and reticular IgE staining in germinal centers [1]. Most of these findings appear to be non-specific; Warthin–Finkeldey cells are seen in measles and HIV infections, and reticular IgE staining in germinal centers is reported in IgG4-RD and atopic dermatitis [8,9]. However, eosinophilic microabscesses in germinal centers may be specific for KD; this finding has not been reported in other diseases. In our case, the eosinophilic infiltrate suggested KD as a differential diagnosis, although eosinophilic microabscesses and germinal centers involving eosinophils were absent.

As a diagnostic modality for KD, FNAC may contribute to its diagnosis. A histological evaluation may be omitted if FNAC confirms polymorphous lymphoid cells with eosinophils and Warthin–Finkeldey cells [10,11]. In our case, FNAC was not diagnostic for KD due to the lack of eosinophils and Warthin–Finkeldey cells; the samples obtained by FNAC likely represented reactive lymphoid follicles.

The histological diagnosis in our case was complicated; the many squamoid nests and cysts in the lesion excluded salivary gland epithelial tumors from the diagnosis, including lymphadenoma and mucoepidermoid carcinoma. In addition, the presence of abundant lymphoid tissue increased the number of differential diagnoses: MALT lymphoma, Sjögren syndrome, HIV infection, and IgG4-related disease. Thus, a clinicopathological evaluation was required for diagnosis. The squamous nests and cysts appeared to show squamous metaplasia of the epithelium of the parotid glands or ducts, probably induced by KD-related inflammation. Squamous metaplasia is observed in necrotizing sialometaplasia of parotid glands; necrotizing sialometaplasia was unlikely for our case because of the absence of lobular architecture of the epithelium, lobular infarction, and extravasated mucin. Furthermore, angiolymphoid hyperplasia with eosinophilia (ALHE), which may overlap with KD, was also suggested as a differential diagnosis. ALHE was ruled out because salivary gland involvement, peripheral blood eosinophilia, and elevated IgE levels are rare in ALHE, and IgE-positive germinal centers are not seen in ALHE [12], [13], [14]. Peripheral blood eosinophilia and high levels of IgE are typical features of KD; however, these findings are also observed in IgG4-RD [12]. Clinicopathological overlap between IgG4-RD and KD has been reported [9]; this was evident in the present case because our patient showed elevated levels of serum IgG4, the presence of >10 IgG4-positive cells/high power field, and a >40% IgG4/IgG ratio. However, the lack of dense lymphoplasmacytic infiltrates, striform fibrosis, and obliterative phlebitis suggested IgG4-RD, which was unlikely for our case.

Recently, HPyV6 was shown to be a possible cause of KD and ALHE [3,4]; HPyV6 DNA has been detected in 80% of KD and ALHE cases [3]. However, the presence of HPyV6 DNA is not specific to KD and ALHE; HPyV6 DNA has also been detected in Kikuchi disease and dermatopathic lymphadenopathy [3]. Our case was the sixth or seventh case of KD that evaluated the HPyV6 DNA of the lesion. Hashida et al. reported that 4 of 5 cases of KD tested positive for HPyV6, and all HPyV6-positive cases resulted in the recurrence of KD within 2 years; HPyV6 DNA was detected in all recurrent lesions [3]. In contrast, the HPyV6-negative case did not recur during the same follow-up period [3]. Although HPyV6-negative KD may show a better clinical course than virus-positive KD, the clinical significance of HPyV6-positive KD lesions warrants further investigation due to the small number of known case studies. Fan et al. reported that all 6 patients with KD treated only by surgery experienced KD recurrence within one year after surgery [15]. Zhang et al. reported that 13 patients (46.4%) with KD relapsed over 1 to 13 years (median: 8.5 years) [16]. Thus, long-term follow-up of the patient is recommended.

In addition, our patient had a history of atopic dermatitis. HPyV6-positive KD lesions may be related to atopic dermatitis. Although reported cases of patients with KD accompanied by atopic dermatitis are rare [17,18], KD and atopic dermatitis may be associated with each other since they are diseases with hyperIgE immunoglobulinemia. HPyV6 infection levels are significantly higher in skin swabs from Japanese patients with atopic dermatitis than in healthy controls [19]. Thus, HPyV6 may be related to allergic skin inflammation, and HPyV6 may reach the internal organs where the virus-associated inflammation may be recognized as KD.

In summary, we present the case of a young woman with KD with a unilateral and solitary HPyV6-negative parotid gland lesion. The KD lesion clinically mimicked a swollen neck lymph node and pathologically mimicked a parotid gland tumor with squamous nests and cysts of various sizes. However, the presence of an epithelial component in the KD lesion made diagnostic imaging and pathological diagnosis difficult.

## Patient consent

Written informed consent was obtained from the patient for the publication of this case report.
